# Electrical impedance tomography measured at two thoracic levels can visualize the ventilation distribution changes at the bedside during a decremental positive end-expiratory lung pressure trial

**DOI:** 10.1186/cc10354

**Published:** 2011-08-11

**Authors:** Ido G Bikker, Carsten Preis, Mahamud Egal, Jan Bakker, Diederik Gommers

**Affiliations:** 1 Department of Intensive Care Medicine, Erasmus MC, 's-Gravendijkwal 230, NL-3015GE Rotterdam, The Netherlands; 2 Department of Anesthesiology, Erasmus MC, 's-Gravendijkwal 230, NL-3015GE Rotterdam, The Netherlands

**Keywords:** electric impedance, mechanical ventilation, positive-pressure respiration, atelectasis, critical care, humans

## Abstract

**Introduction:**

Computed tomography of the lung has shown that ventilation shifts from dependent to nondependent lung regions. In this study, we investigated whether, at the bedside, electrical impedance tomography (EIT) at the cranial and caudal thoracic levels can be used to visualize changes in ventilation distribution during a decremental positive end-expiratory pressure (PEEP) trial and the relation of these changes to global compliance in mechanically ventilated patients.

**Methods:**

Ventilation distribution was calculated on the basis of EIT results from 12 mechanically ventilated patients after cardiac surgery at a cardiothoracic ICU. Measurements were taken at four PEEP levels (15, 10, 5 and 0 cm H_2_O) at both the cranial and caudal lung levels, which were divided into four ventral-to-dorsal regions. Regional compliance was calculated using impedance and driving pressure data.

**Results:**

We found that tidal impedance variation divided by tidal volume significantly decreased on caudal EIT slices, whereas this measurement increased on the cranial EIT slices. The dorsal-to-ventral impedance distribution, expressed according to the center of gravity index, decreased during the decremental PEEP trial at both EIT levels. Optimal regional compliance differed at different PEEP levels: 10 and 5 cm H_2_O at the cranial level and 15 and 10 cm H_2_O at the caudal level for the dependent and nondependent lung regions, respectively.

**Conclusions:**

At the bedside, EIT measured at two thoracic levels showed different behavior between the caudal and cranial lung levels during a decremental PEEP trial. These results indicate that there is probably no single optimal PEEP level for all lung regions.

## Introduction

Electrical impedance tomography (EIT) is a promising new tool for bedside monitoring of regional lung ventilation and changes in regional and global lung volume [1-3]. EIT is a technique based on the injection of small electrical currents and voltage measurements using electrodes on the skin surface. Cross-sectional images are generated from these measurements, representing impedance change in a 5- to 10-cm-wide slice of the thorax. It is a radiation-free, noninvasive, portable lung imaging technique.

EIT can be performed at different craniocaudal levels of the thoracic cage, but the caudal thoracic level just above the diaphragm is of particular importance because atelectasis due to mechanical ventilation can be expected most at this level. On the basis of computed tomography (CT), it is known that the dorsal and dependent lung regions are most susceptible to alveolar collapse and recruitment [4]. Therefore, these lung regions would benefit most from individualized positive end-expiratory pressure (PEEP) settings, and consequently most EIT studies have been performed at the caudal thoracic level in the dependent lung regions [5,6]. However, conflicting results have been found between regional lung parameters measured with EIT [6-8] and those measured with global lung parameters, such as compliance and end-expiratory lung volume (EELV) [9,10]. In a previous study, we found that tidal impedance variation measured at one thoracic level was not equal to tidal volume, and we speculated that this might be due to derecruitment [9]. As derecruitment occurs most prominently in the dorsal lung regions above the diaphragm, the relation between global lung parameters and the results of regional lung parameters measured with EIT would be dependent on the measurement location. However, the relation between ventilation distributions in the craniocaudal axis has not yet been studied with EIT. Therefore, in this study, we evaluated whether ventilation distribution measured by EIT, as well as the relation between EIT regional compliance and global dynamic compliance, differs between cranial and caudal thoracic levels in response to decremental PEEP steps.

## Materials and methods

Following approval by the local institutional human investigations committee, patients were enrolled in this study after we obtained their written informed consent. The study data were collected during an ongoing study which investigated lung condition and the response to PEEP before, during and after cardiothoracic surgery. The study population consisted of 12 mechanically ventilated patients in a cardiothoracic ICU. Exclusion criteria for participation in the study were pneumothorax, severe airflow obstruction due to chronic obstructive pulmonary disease (defined as forced expired volume in one second or vital capacity below predicted value minus two standard deviations), thoracic deformations and severe cardiovascular instability.

### Study protocol

After the surgical procedure and after the patient's arrival at the cardiothoracic ICU, measurements were taken during the warm-up period following the surgical procedure. During this period, all patients were fully sedated and ventilated in pressure-controlled mode without any signs of spontaneous breathing activity. Prior to this protocol, hypovolemia was corrected by the anesthesiologist. All patients were in the supine position.

**Figure 1:**
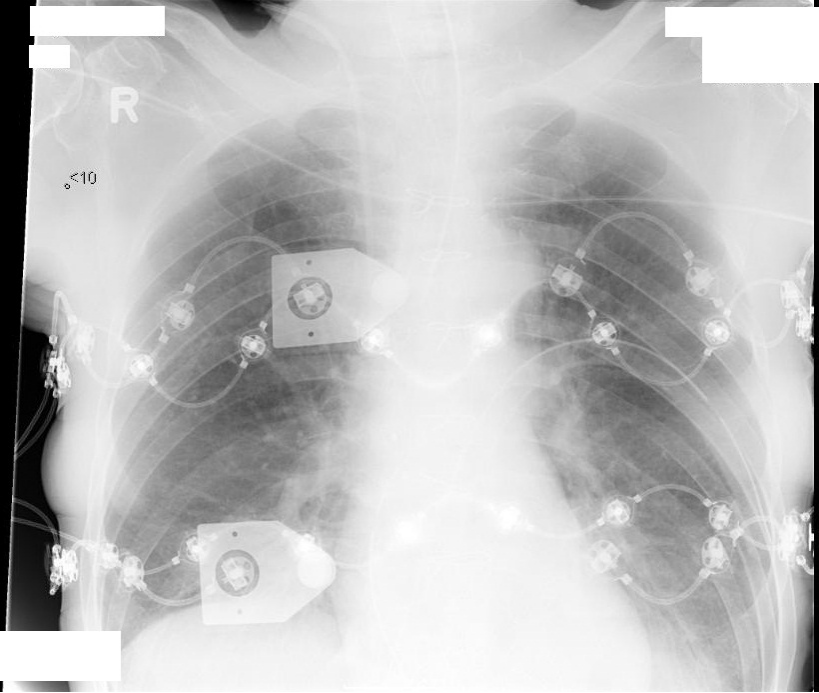
**Chest X-ray of one of the studied patients showing the cranial (upper) and caudal (lower) electrical impedance tomography belt**. X-ray taken at 5 cm H_2_O positive end-expiratory pressure (PEEP).

After a stabilization period of 15 minutes, 2 silicone EIT belts, each with 16 integrated cardiographic electrodes, were placed around the thoracic cage (Figure 1). In all patients, EIT was measured at a caudal level and a cranial thoracic level during decremental PEEP steps according to the study protocol. One belt was placed at the highest possible thoracic level, just under the armpits, at the third or fourth intercostal space. The second belt was placed just below the nipples at the sixth or seventh intercostal space. The belts were connected to a single EIT device (EIT evaluation kit 2; Dräger, Lübeck, Germany) in an alternating fashion. EIT data were generated by application of a small alternating electrical current of 5 mA at 50 kHz.

PEEP was increased to 15 cm H_2_O, and a recruitment maneuver with peak inspiratory pressure (PIP) of 40 cm H_2_O and PEEP of 20 cm H_2_O was applied for 40 seconds. After a steady state of 15 minutes at 15 cm H_2_O PEEP, PEEP was decreased stepwise from 15 to 10 cm H_2_O, then to 5 cm H_2_O and, if clinically acceptable, to 0 cm H_2_O. Each PEEP was applied for 15 to 20 minutes. The driving pressure (PIP minus PEEP) was kept constant at all used PEEP levels. Before the end of each PEEP level, EIT was measured at both levels during a two-minute period, and hemodynamic and ventilatory parameters were recorded. Pressure-controlled ventilation was used specifically to enable the evaluation of regional compliance independently of other lung regions. Dynamic compliance was calculated by dividing expiratory tidal volume measured at the Y-piece of the Engström Carestation ventilator (GE Healthcare, Madison, WI, USA) by the constant driving pressure set for each patient. In addition, arterial blood gas analysis was performed (i-STAT MN300 System; Abbott Point of Care, Inc., Princeton, NJ, USA) to calculate the ratio of partial pressure of oxygen to fraction of inspired oxygen at each PEEP level.

### EIT analysis

**Figure 2:**
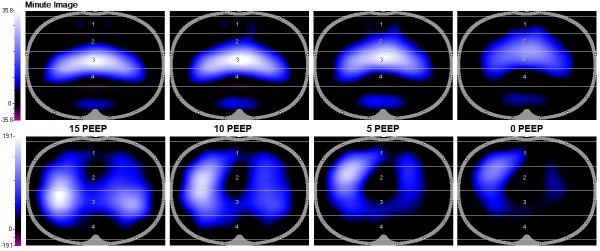
**Images of ventilation distribution at a cranial (top row) and caudal (bottom row) thoracic lung levels at the different decremental positive end-expiratory pressure (PEEP) steps in a representative patient**. Images were obtained by averaging tidal impedance distribution over one minute. The regions of interest (ROIs) used in the present study are displayed at both levels and defined according to the anatomical position of lung tissue. Nondependent lung regions: 1 and 2. Dependent lung regions: 3 and 4.

EIT data were stored and analyzed offline on a personal computer. The EIT scans consist of images showing impedance with a 32 × 32 color-coded matrix relative to the lowest impedance recorded during the PEEP trial (relative Δ*Z*). The difference between relative Δ*Z *at the end of inspiration and expiration is defined as tidal impedance variation. Tidal impedance variation was visualized on functional EIT (fEIT) images, which showed tidal impedance variation per pixel (32 high × 32 width matrix) averaged over one minute (Figure 2).

EIT regional compliance was calculated by dividing the tidal impedance variation by the driving pressure [11]. The concept of regional compliance measured with EIT is possible because of the proportional relation between local impedance and local volume changes [7]. Pressure-controlled ventilation was used specifically to enable the evaluation of regional compliance independently of other lung regions.

For analysis of the regional distribution of ventilation, the EIT images were subdivided into two symmetrical nonoverlapping layers from the ventral to dorsal orientation, defined as regions of interest (ROIs). This was done in a different fashion for the measurements recorded at the cranial and caudal thoracic levels based on the anatomical position of lung tissue (Figure 2). At the caudal level, each of these ROIs, from line 3 to 30, was 14 × 32 pixels. At the cranial level, each of these ROIs, from line 3 to 22, was 10 × 32 pixels. In addition, to minimize influences from the diaphragm, we selected smaller upper and lower ROIs of each of the four horizontal lines (4 × 32 pixels) at the center of the above-described upper and lower ROIs. This was done at both the caudal and cranial levels.

Distribution of EIT tidal ventilation in a ventral to dorsal direction was expressed according to the center of gravity index [12]. Change in global tidal impedance was divided into two equal ROIs, dorsal and ventral, each accounting for 50% of the EIT image. The center of gravity was calculated by dividing the dorsal tidal impedance variation by the total tidal impedance variation.

Volume changes were calculated by dividing changes in end-expiratory lung impedance by the ratio of tidal impedance variation to tidal volume. As this ratio differs between PEEP levels, it was averaged between successive PEEP levels to calculate lung volume changes between successive PEEP levels (for example, from baseline 5 to 15 cm H_2_O or from 15 to 10 cm H_2_O).

### Statistical analysis

Statistical analysis was performed with the GraphPad version 5.0 software package (GraphPad Software, Inc., San Diego, CA, USA). Unless stated otherwise, results are expressed as means ± standard deviations for normally distributed data and medians ± interquartile ranges for nonnormally distributed data. The Shapiro-Wilk normality test was used to evaluate the distribution of all data. The Friedman test for the one-way analysis of variance for repeated measures was used to study the change at the decremental PEEP steps of the center of gravity index, ratio of tidal impedance variation to tidal volume, dynamic compliance and arterial oxygenation. For all comparisons, *P *< 0.05 was considered significant.

## Results

Table 1 presents the physiologic and demographic data of the study population. Dynamic compliance and arterial oxygenation data are shown in Table 2. Figure 2 shows the fEIT images averaged over one minute in one representative patient at 15, 10, 5 and 0 cm H_2_O PEEP at both thoracic levels. During the decremental PEEP trial, the area of ventilation increased at the cranial level but decreased at the caudal level (Figure 2).

**Table 1:** Patient characteristics^a^

Characteristics	Data
Number of patients	12
Females/males	3/9
Age (years)	69.6 ± 9.4
Height (m)	1.72 ± 0.08
Body weight (kg)	78.8 ± 12.3
Baseline PEEP (cm H_2_O)	5.0 ± 0.0

^a^PEEP, positive end-expiratory pressure. Data are means ± standard deviations unless otherwise indicated.

**Table 2:** Arterial oxygenation, compliance and ventilator settings during decremental PEEP steps^a^

	PEEP (cm H_2_O)				
	
Measurement	Baseline 5	15	10	5	0
Compliance (mL/cm H_2_O)	52.9 ± 9.6	60.2 ± 9.2	63.0 ± 8.6	57.9 ± 7.8	41.2 ± 7.4^b^
PaO_2_/FiO_2 _ratio (kPa)	39.9 ± 15.7^b^	64.5 ± 13.7	61.9 ± 14.8	47.5 ± 14.4^c^	28.8 ± 11.2^d^
Ventilatory mode	PCV	PCV	PCV	PCV	PCV
Inspiratory-to-expiratory ratio	1:1	1:1	1:1	1:1	1:1
Driving pressure (cm H_2_O)	10 ± 2	10 ± 2	10 ± 2	10 ± 2	10 ± 2
Respiratory rate (breaths/minute)	15 ± 1	15 ± 1	15 ± 1	15 ± 1	15 ± 1
Tidal volume (mL)	491 ± 54	591 ± 120	615 ± 103	565 ± 91	403 ± 88

^a^PaO_2_/FiO_2 _ratio: ratio of partial pressure of oxygen to fraction of inspired oxygen; PCV: pressure-controlled ventilation; PEEP, positive end-expiratory pressure. BL: Baseline. Data are means ± standard deviations unless otherwise indicated. Significance compared to 15 cm H_2_O: ^b ^< 0.01, ^c ^< 0.05, ^d ^< 0.001.

**Figure 3:**
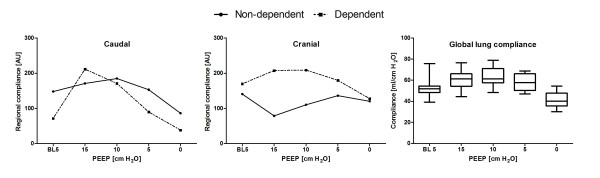
**Dynamic compliance (right) and regional compliance measured at a caudal (left) and cranial (middle) thoracic lung levels at dependent orientation (dorsal) and nondependent orientation (ventral) regions of interest (ROIs)**. Regional compliance was calculated by dividing the electrical impedance tomography (EIT) tidal impedance variation by the applied driving pressure. Box and whisker plots: 95%, 75%, 50%, 25% and 5%. AU: arbitrary units.

The regional compliance of the two ROIs at both thoracic levels is shown in Figure 3 at each decremental PEEP step. The total regional compliance of both ROIs decreased at the caudal level during the decremental PEEP trial, whereas it increased initially at the cranial level after PEEP was lowered from 15 to 10 cm H_2_O. At the caudal level, regional compliance decreased rapidly during the decremental PEEP steps. At the cranial level, the tidal impedance variation increased in the nondependent ROI and decreased in the dorsal ROI during the decremental PEEP steps.

**Figure 4:**
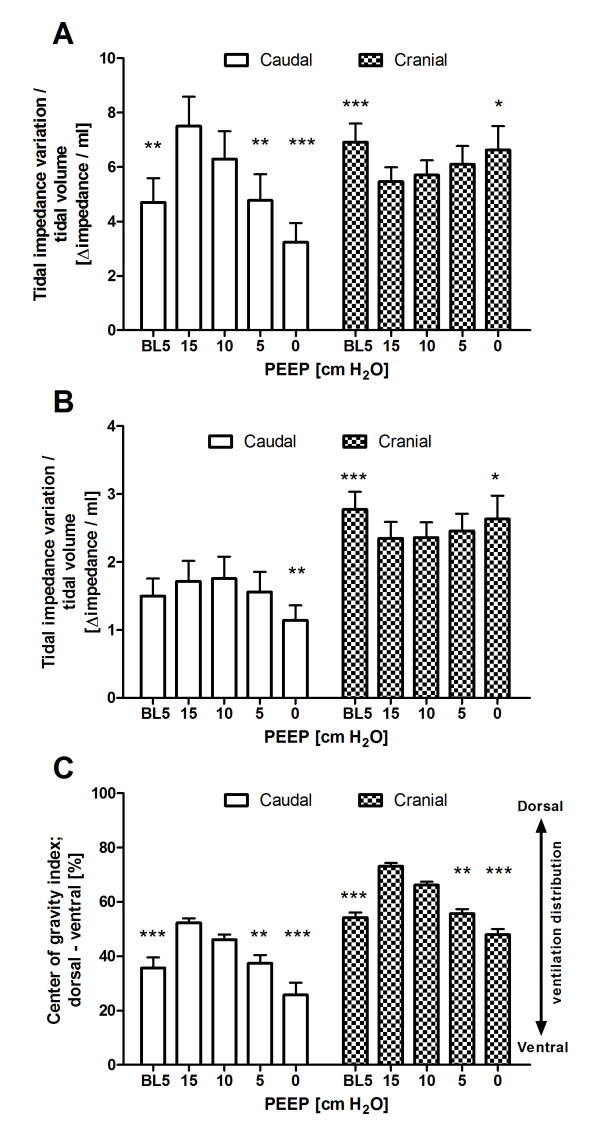
**Tidal impedance variation divided by tidal volume obtained from (A) regions of interest (ROIs) to include all anatomical lung tissue and from ROIs selected to minimize caudal diaphragmatic influence (ROI: 4 × 32 pixels) (B)**. **(C) **Center of gravity index. Measurements were taken during decremental positive end-expiratory pressure (PEEP) steps at the caudal and cranial thoracic levels. Data are as means + standard errors of the mean. Significance compared to 15 cm H_2_O positive end-expiratory pressure (PEEP): *P < 0.05, **P < 0.01, ***P < 0.001.

Both the tidal impedance variation and the tidal volume decreased at the decremental PEEP steps, but the ratio of tidal impedance variation divided by tidal volume decreased at the caudal level and increased at the cranial level during the decremental PEEP trial (Figure 4A). In addition, the center of gravity index decreased at both lung levels during the decremental PEEP trial, which means that tidal impedance variation decreased at the dorsal lung region and decreased less, or even increased, at the ventral lung regions after lowering the PEEP (Figure 4C).

The ratio of tidal impedance variation divided by tidal volume from the small ROIs, to minimize diaphragm influence, is shown in Figure 4B. It also decreased at the caudal level and increased at the cranial level during the decremental PEEP trial, but the changes were smaller compared to the original ROIs, which were designed to cover all anatomical lung tissue.

**Figure 5:**
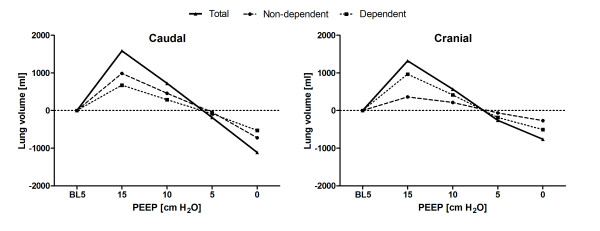
**End-expiratory lung volume (EELV) calculated from end-expiratory impedance changes**. Values are relative to baseline 5 cm H_2_O positive end-expiratory pressure (PEEP), which was set at 0.

Lung volume changes compared to baseline are shown in Figure 5, calculated from both caudal and cranial EIT measurement levels. The rate of EELV decrease was nonsignificantly different between the caudal and cranial levels. At the caudal level, the rate increased, whereas at the cranial level, the rate of lung volume loss decreased during the decremental PEEP steps.

## Discussion

In this study, the center of gravity index measured with EIT decreased at both the cranial and caudal thoracic levels during a decremental PEEP trial, whereas tidal impedance variation divided by tidal volume decreased at the caudal level and increased at the cranial level. This indicates that during a decremental PEEP trial, ventilation distribution not only shifted from the dorsal to ventral direction, but also from the caudal to cranial direction during mechanical ventilation in post-cardiac surgery patients.

To the best of our knowledge, this study is the first to investigate ventilation distribution using EIT at multiple levels in mechanically ventilated patients at the bedside. Both the tidal impedance variation and the tidal volume decreased at the decremental PEEP steps, but the ratio of tidal impedance variation divided by tidal volume decreased at the caudal level and increased at the cranial level during the decremental PEEP trial (Figure 4). Multiple CT studies have shown a highly correlated linear relation between local air and impedance distribution [6-8], suggesting that local ventilation decreases more at the caudal level that at the cranial level. It becomes clear that the dorsal-dependent lung regions just above the diaphragm are the areas with most potential for lung collapse and loss of ventilation at lower PEEP levels. This particular area was measured with EIT with the caudal belt placed just below the nipples at the sixth or seventh intercostal space. However, at this level, the effects of the diaphragm's entering into the middle of the EIT eclipse also can contribute to the major decrease in tidal impedance variation. When smaller ROIs were selected, tidal impedance variation still decreased, but to a lesser degree. In addition, we have shown that the center of gravity decreased during the decremental PEEP trial, which means that tidal impedance variation decreased more at the dorsal lung region and decreased less, or even increased, at the ventral lung region after PEEP was lowered (Figures 3 and 4). The impedance shift from the dorsal to ventral lung region during the decremental PEEP steps was due to the onset of more collapse at the dorsal site than at the ventral site at the PEEP levels applied, as shown by the individual impedance changes in the different ROIs (Figure 3). This finding is in agreement with the common understanding that ventilation distribution is gravity-dependent, as shown by CT [4]. At the cranial level, tidal impedance variation even increased after the PEEP level was lowered from 15 to 10 cm H_2_O and increased further after the PEEP level was lowered in the most ventral ROIs (Figure 3). This finding can be explained by the reduction in overdistention in the nondependent lung parts in the upper part of the lung. This means that applying a PEEP level of 15 cm H_2_O was too high for the cranial site but not for the caudal site, indicating that the optimal PEEP level is different for these sites.

Regional compliance, as calculated by regional impedance changes divided by the pressure amplitude [11,12], showed different ventilation distribution behavior in response to the decremental PEEP steps at both studied lung levels (Figure 3). In our study, because driving pressure was kept constant in each patient during the decremental PEEP steps, the tidal impedance variation can be regarded as regional compliance. In this study, the behavior of the global dynamic compliance was similar to the regional compliance at the cranial level but not at the caudal level (Figure 3). Both global compliance and total regional compliance at the cranial level increased initially after lowering PEEP from 15 to 10 cm H_2_O PEEP, whereas both global and total regional compliance decreased during the next PEEP steps (Figure 4). For decremental PEEP steps, this finding is explained by the classical finding that compliance increases as a result of the release of overdistended alveoli until collapse increases and lowers the compliance. This has been described in detail previously by Suarez-Sipmann *et al*. [13]. Inspection of the individual regional compliance behavior of the different ROIs showed different PEEP levels at which the regional compliance is maximal (Figure 3). This finding means that there is no single optimal PEEP for the whole lung. This principle was previously proven with pressure impedance curves in lavaged pigs by Kunst *et al*. [14] and explained in an excellent review by Hickling [15]. In those studies, multiple pressure impedance curves in the dorsal to ventral direction were generated from an inspiratory pressure-volume curve, and different lower and upper inflection points were found. However, only the dorsal to ventral direction was studied. In the present study, we have also shown, on the basis of EIT in patients at the bedside, that this is true in the caudal to cranial direction. Our study results are also in agreement with a recent CT study of acute respiratory distress syndrome patients by Caironi *et al*. [16], who also found different optimal PEEP values in the craniocaudal direction to counteract the different superimposed pressures calculated by CT. The different superimposed pressure levels on each lung section are one of the most important explanations for the difference in optimal PEEP levels, as we found in the present study. Especially in the dorsal and caudal lung regions, the weight of the heart and edematous lung tissue in respiratory failure will cause atelectasis and surfactant depletion.

We calculated EELV changes at both the caudal and cranial lung levels. Both levels showed a comparable pattern, but the rate of lung volume loss differed nonsignificantly during the decremental PEEP steps. This finding could be explained by increased lung volume due to atelectasis at the basal part of the lung at lower PEEP levels. However, calculation of lung volume changes on the basis of regional EIT measurement calibrated with a global lung parameter (tidal volume) does have limitations. In our former study, we showed that EIT-based lung volume calculation does not correlate well with lung volume measured using a multibreath nitrogen washout technique [9]. Also, the use of a global lung parameter (for example, tidal volume) with unpredictable distribution limits these calculations. Furthermore, this study shows that ventilation distribution during PEEP changes can vary not only in a ventral to dorsal direction but also in a cranial to caudal direction. Therefore, calculations made using regional lung impedance data can be used only as an indication of global lung volume changes. In addition, when calculating lung volume during PEEP steps on the basis of EIT and tidal volume, it is necessary to calculate the mean impedance to volume ratio from the high and low PEEP levels, as this ratio is not constant when PEEP is changed.

The present study was performed in patients after cardiothoracic surgery because these patients demonstrate a uniform lung condition and are highly responsive to PEEP. We earlier showed that using a PEEP strategy combined with recruitment maneuvers in patients after cardiothoracic surgery, lung volume could be preserved, inflammatory reaction could be reduced and increase in right ventricular afterload could be minimized [17-19]. Several factors are known to affect lung function postoperatively, including cardiopulmonary bypass (which is known to release inflammatory mediators), mechanical ventilation and mechanical compression of the left lung during the surgical procedure itself [20].

EIT-based measurements taken at multiple thoracic levels have some inherent methodological issues. Because of the position and shape of the thoracic cage and lungs, not all lung regions can be studied. In this study, we positioned the cranial EIT belt at the highest possible level, just under the armpits, taking measurements at a lung level just above the hilus (Figure 1). The most caudal belt was positioned just under the nipples at the sixth or seventh intercostal space, so that measurements were taken just above the diaphragm (Figure 1). At this level, the diaphragm could enter the EIT eclipse and thereby change the amount of lung volume during PEEP steps. This would be especially true in critically ill patients who are known to have reduced EELV [21] and at high and low PEEP levels. In our study, we tried to avoid these effects by selecting smaller ROIs away from the middle of the EIT eclipse, where the effects of the diaphragm are most likely. The tidal impedance variation to tidal volume ratio still decreased, but it was lower compared to the ROIs covering all anatomical lung tissue (Figure 4B). Also, the heart moves upward and sideways as PEEP decreases, thereby taking more space within the left portion of the electrode plane, as can be anticipated from Figure 2. Furthermore, the EIT device used in the present study measures an eclipse with a central diameter of 5 to 10 cm. Moreover, the distance between the two belts was about 10 cm, indicating that almost all of the accessible lung tissue for EIT was measured with both belts. Although techniques for three-dimensional (3D) measurement of impedance are under development [22-24], they are not yet available in clinical practice. On the basis of the findings of the present study, measurement of ventilation distribution in the cranial to caudal direction with 3D EIT would provide additional information, although the most caudal region would still provide the most clinically relevant information in the ICU population.

## Conclusions

On the basis of the findings of this study, ventilation distribution shifts not only from the dorsal to ventral region but also from the caudal to cranial level during decremental PEEP. Also, in the mechanically ventilated patients in our study, the PEEP level at which regional compliance was maximal differed for the dependent and nondependent lung regions as well as for the caudal and cranial lung levels, suggesting that no single optimal PEEP exists for all lung regions. The clinical implications of these findings are that, during clinical PEEP titration, one has to realize that the results are dependent on the measurement location. Also, a lung-monitoring system (for example, EIT) at the bedside is a prerequisite to finding the optimal balance in finding the best ventilatory settings. Further, EIT measurements taken at two thoracic levels, that is, the caudal and cranial parts of the lung, provides additional information for monitoring ventilation distribution during mechanical ventilation in the whole lung.

## Key messages

• Ventilation distribution changes measured using EIT during a PEEP trial were comparable to CT, but the EIT monitoring measurements were taken at the bedside.

• The optimal PEEP level differs between lung regions: from the cranial to caudal region and from the ventral to dorsal region.

• To monitor ventilation distribution during a PEEP trial, EIT measurements should be taken in the caudal and cranial parts of the lung.

• If EIT is measured at a caudal lung level during high and low PEEP steps, the results can be influenced by the diaphragm's entering the EIT eclipse.

## Abbreviations

Δ*Z*: impedance change; CT: computed tomography; EELV: end-expiratory lung volume; EIT: electrical impedance tomography; fEIT: functional electrical impedance tomography; PEEP: positive end-expiratory pressure; ROI: region of interest.

## Competing interests

The authors declare that they have no competing interests.

## Authors' contributions

IB and CP carried out the data acquisition, data processing, data analysis and statistical analysis and participated in drafting the manuscript. ME participated in the data acquisition, data processing and statistical analysis. DG and JB participated in data acquisition and drafting the manuscript. All authors read and approved the final version of the manuscript.
